# Health Risk Assessment of Heavy Metals in Soils from Witwatersrand Gold Mining Basin, South Africa

**DOI:** 10.3390/ijerph13070663

**Published:** 2016-06-30

**Authors:** Caspah Kamunda, Manny Mathuthu, Morgan Madhuku

**Affiliations:** 1 Center for Applied Radiation Science and Technology, North West University (Mafikeng), Private Bag X2046, Mmabatho 2735, South Africa; Manny.Mathuthu@nwu.ac.za; 2 iThemba LABS, National Research Foundation, Private Bag X11, Wits 2050, South Africa; madhuku@tlabs.ac.za

**Keywords:** ICP-MS, heavy metal, hazard index, average daily intake, carcinogenic risk, non-carcinogenic risk

## Abstract

The study evaluates the health risk caused by heavy metals to the inhabitants of a gold mining area. In this study, 56 soil samples from five mine tailings and 17 from two mine villages were collected and analyzed for Asernic (As), Lead (Pb), Mercury (Hg), Cadmium (Cd), Chromium (Cr), Cobalt (Co), Nickel (Ni), Copper (Cu) and Zinc (Zn) using ICP-MS. Measured concentrations of these heavy metals were then used to calculate the health risk for adults and children. Their concentrations were such that Cr > Ni > As > Zn > Cu > Co > Pb > Hg > Cd, with As, Cr and Ni higher than permissible levels. For the adult population, the Hazard Index value for all pathways was found to be 2.13, making non-carcinogenic effects significant to the adult population. For children, the Hazard Index value was 43.80, a value >>1, which poses serious non-carcinogenic effect to children living in the gold mining area. The carcinogenic risk was found to be 1.7 × 10^−4^ implying that 1 person in every 5882 adults may be affected. In addition, for children, in every 2725 individuals, 1 child may be affected (3.67 × 10^−4^). These carcinogenic risk values were both higher than acceptable values.

## 1. Introduction

Heavy metals are a common occurrence in the environment and have resulted in human exposure for the entire history of mankind. However, anthropogenic activities such as mining have resulted in elevated levels of these contaminants in the environment. By definition, any toxic metal may be called a heavy metal, irrespective of its atomic mass or density. The classification includes some metalloids, transition metals, basic metals, lanthanides and actinides and metals of groups III to V of the periodic table [1]. Examples include As, Pb, Hg, Cd, Cr, Co, Ni, Cu, Zn, Se, Al, Cs, Mn, Mo, Sr, U, Be and Bi [2].

Some metals are essential to life and play irreplaceable roles as sources of vitamins, and minerals in the functioning of body organs. All living organisms require varying amounts of metals, but become toxic at higher concentrations [3]. Other metals have no useful role in the human physiology. Examples of such elements are arsenic, lead and mercury. They may be toxic even at low levels of exposure. Once absorbed by the body, heavy metals continue to accumulate in vital organs like the brain, liver, bones, and kidneys, for years or decades causing serious health consequences [4]. Arsenic, lead and mercury are the first, second and third hazards on the priority list of heavy metal pollutants as designated by the United States Agency for Toxic Substances and Disease Registry [5].

Arsenic, for instance, is regarded a human carcinogen from extremely low levels of exposure [6]. Acute exposure to arsenic compounds may cause nausea, vomiting, abdominal pain, muscle cramps and diarrhoea [7] while chronic exposure is associated with peripheral nerve damage causing diabetes [8]. Pb on the other hand, is regarded as a human mutagen and probable carcinogen [9]. It induces renal tumours, and also disturbs the normal functioning of kidneys, joints, reproductive and nervous systems [10].

The acute ingestion of inorganic Hg potentially causes gastrointestinal disorders, diarrhoea, and haemorrhage [8]. Repeated and prolonged exposure may seriously affect the kidneys, liver and skin. Cd is known to be toxic even at low concentrations and is also regarded as a probable carcinogen. Severe exposure to Cd may result in pulmonary effects such as bronchiolitis, emphysema, and alveolitis [4]. Cd can also result in bone fracture, kidney dysfunction, hypertension and even cancer [11]. Arthritis, diabetes, anaemia, cardiovascular disease, cirrhosis, reduced fertility, headaches and strokes are some of its odd long term effects.

Whereas chromium (III) is an essential element [9], chromium (VI) compounds are known to be mutagenic and carcinogenic. Breathing high levels of chromium (VI) may cause asthma and shortness of breath. Long term exposure may cause damage to the liver and kidneys. Ni on the other hand is known to cause cancer, both oral and intestinal. It also causes depression, heart attacks, haemorrhages and kidney problems [7]. Excessive intake of Zn and Cu may cause non-carcinogenic effects on human health, even though they are essential to human life [12]. Cu surplus had been associated with liver damage while Zn may cause impairment of growth and reproduction [13].

The Witwatersrand Gold Mining Basin is the world largest that covers an area of 1600 km^2^. Mining activities have led to a legacy of some 400 km^2^ of mine tailings [14]. Gold mine tailings in the study area constitute a major source of heavy metal pollution to the environment. Major mechanisms through which heavy metals can be transported from mine tailings are the atmosphere, ground water sources and surface water body. Their accumulation in soil, air, water and edible parts of plants represents a direct pathway for their incorporation into the human food chain. In the study area, air pollution has been reported as a result of soil being blown by wind from mine tailings and the surroundings [14]. Although no health risk studies have been carried out in the mining area, children of community members have often been reported sick due to chest pains, tuberculosis diarrhoea, cough and itchy skin [15]. Liefferink [16] also observed, on frequent occasions, children playing with soil and young mothers eating salt crusts from mine tailings. Although this has been the case, information about heavy metal contamination and their risks is lacking. Therefore, the study aimed to measure the concentration levels of heavy metals in soils from the study area, and to estimate the health risks on the residents. In this study, nine heavy metals, namely As, Pb, Hg, Cd, Cr, Co, Ni, Cu and Zn were included in the measurement.

## 2. Materials and Methods

### 2.1. Study Area

**Figure 1:**
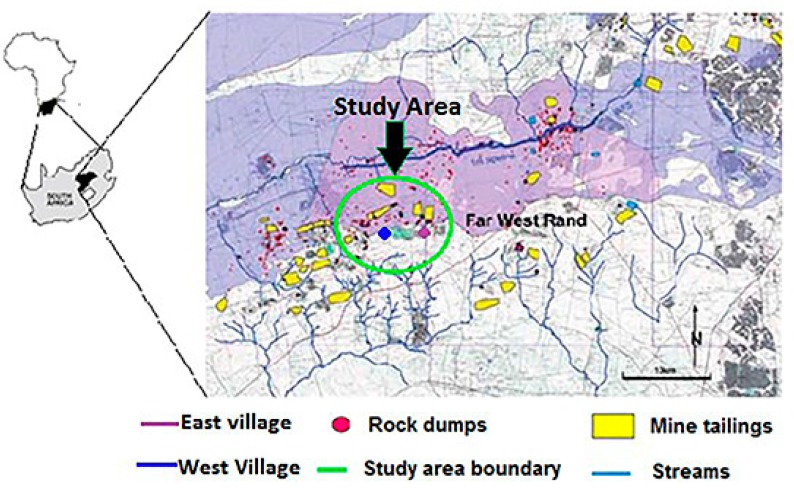
The study area: Part of the Witwatersrand gold mining basin.

The study area is a gold mining area situated some 70 km west of Johannesburg in the Gauteng Province of South Africa. It lies between 26°18’ S–26°26’ S latitude and 27°23’ E–27°31’ E longitude. Gold exploration in the area dates back to 1898 and mining started from 1945 to date. Geographically the area, which is approximately 86 km^2^ is located in the West Wits line (Far West Rand) Goldfield of the Witwatersrand Gold Mining Basin. The study area is densely populated with informal settlements residing close to gold mine tailings. The topography of the area is relatively flat and the vegetation is largely grassland. The climate is temperate, with temperatures averaging 24 °C in summer and 13 °C in winter. Annual rainfall is about 750 mm [17]. Figure 1 shows the map of the study area.

### 2.2. Sampling and Sample Preparation

A total of 56 soil samples from five mine tailings and 17 from two mine villages representative of the gold mining area were collected with a steel auger at a depth of 5 cm. At each sampling location, five replicate samples were collected within a 2 m × 2 m grid, thoroughly mixed to obtain a homogenous sample, out of which 1 kg was packaged in polyethylene bags [18]. All the collected samples were properly marked and identified by their sampling locations using a Global Positioning System (GPS) receiver. The collected soil samples were taken to the laboratory for further processing. A common problem in the analysis of samples for As and Hg is loss during sample preparation. As a precautionary measure, ashing was avoided. At the laboratory, the soil samples were first spread out on a plastic sheet and allowed to air dry for 2–3 days. The samples were then sieved through a 2 mm nylon mesh to obtain a homogenous sample matrix. Close attention was paid to every sample to avoid cross-contamination [17].

### 2.3. Soil Sample Analysis

The soil samples were then analysed for heavy metal elements using Inductively Coupled Plasma-Mass Spectrometry (ICP-MS), model PerkinElmer NexION 300 ICP-MS (PerkinElmer, Waltham, MA, USA). Before ICP-MS was done, soil samples were first digested in a Multiwave 3000 microwave system. Digestion is done in order to dissolve the heavy metals from the soil samples. In this process, 1 g dry weight of each soil sample was accurately measured and mixed with 3 mL of nitric acid and 9 mL of hydrochloric acid in a rotor vessel. 1 mL of hydrogen peroxide also added to the mixture. The mixture was then digested at a temperature of 120 °C for about 25 min and then allowed to cool down for about 15 min. After this, the digested samples were transferred into 100 mL volumetric flasks with 2% HNO_3_. Deionised water was then used to top up to this volume [19]. The digested samples were then allowed to sediment overnight and there after filtered with No. 40 Whatman filter paper in readiness for ICP-MS.

The digested samples were then introduced into the ICP-MS, where the sample components were decomposed into their atomic constituents. TotalQuant method was used together with Perkin Elmer Pure Plus NexION Dual Detector Calibration Solution standard. This method has the advantage of high sensitivity (ng × L^−1^ range), wide linear dynamic detection range and specificity for the accurate detection and quantification of heavy metals. TotalQuant calibration was achieved using 200 µg/L of Al, Ba, Ce, Co, Cu, In, Li, Mg, Mn, Ni, Pb, Tb, U and Zn. The quality of the analytical data was guaranteed by implementing standard quality assurance procedures. Each sample was analysed in duplicates. After every 10 samples, a certified standard and a blank solution were run to check for contamination and drift. All the chemicals and reagents used were of certified analytical grade and procured from Merck (South Africa). The detection limits for As, Pb, Hg, Cd, Cr, Co, Ni, Cu and Zn were 0.015, 0.0003, 0.0003, 0.005, 0.0009, 0.001, 0.006, 0.004 and 0.04 µg/L, respectively. The heavy metal concentrations obtained from the ICP-MS analysis in mg/L were then converted into mg/kg.

## 3. Health Risk Assessment

### 3.1. Theory of Risk Assessment

Human health risk assessment is a process used to estimate the health effects that might result from exposure to carcinogenic and non-carcinogenic chemicals [20]. The risk assessment process is made up of four basic steps: hazard identification, exposure assessment, toxicity (dose-response) assessment, and risk characterization [20].

Hazard Identification basically aims to investigate chemicals that are present at any given location, their concentrations, and spatial distribution. In the study area, As, Pb, Hg, Cd, Cr, Co, Ni, Cu and Zn were identified as possible hazards for the community.

The purpose of exposure assessment is to measure or estimate the intensity, frequency, and duration of human exposures to an environmental contaminant. In the study, exposure assessment was carried out by measuring the average daily intake (*ADI*) of heavy metals earlier identified through ingestion, inhalation and dermal contact by adults and children from the study area. Adults and children are separated because of their behavioural and physiological differences [21].

Dose-response assessment estimates the toxicity due to exposure levels of chemicals. The cancer slope factor (*CSF*, a carcinogen potency factor) and the reference dose (*RfD*, a non-carcinogenic threshold) are two important toxicity indices used. *RfD* values are derived from animal studies using the “No observable effect level” principle. For humans, *RfD* values are multiplied 10-fold to account for uncertainties [22].

Risk characterization predicts the potential cancerous and non-cancerous health risk of children and adults in the study area by integrating all the information gathered to arrive at quantitative estimates of cancer risk and hazard indices [23].

The potential exposure pathways for heavy metals in contaminated soils are calculated based on recommendations by several American publications. *ADI* (mg/kg-day) for the different pathways were calculated using the following exposure Equations (1)–(3) as prescribed by [22].

#### 3.1.1. Ingestion of Heavy Metals through Soil

(1) $$ADI_{ing}=\frac{C\times IR\times EF\times ED\times CF}{BW\times AT\;}$$

where *ADI*_ing_ is the average daily intake of heavy metals ingested from soil in mg/kg-day, *C* = concentration of heavy metal in mg/kg for soil. *IR* in mg/day is the ingestion rate, *EF* in days/year is the exposure frequency, *ED* is the exposure duration in years, *BW* is the body weight of the exposed individual in kg, *AT* is the time period over which the dose is averaged in days. *CF* is the conversion factor in kg/mg.

#### 3.1.2. Inhalation of Heavy Metals via Soil Particulates

(2) $$ADI_{inh}=\frac{C_{s}\times IR_{air}\times EF\times ED}{BW\times AT\times PEF}$$

where *ADI_inh_* is the average daily intake of heavy metals inhaled from soil in mg/kg-day, *C_S_* is the concentration of heavy metal in soil in mg/kg, *IR_air_* is the inhalation rate in m^3^/day, *PEF*, is the particulate emission factor in m^3^/kg. *EF*, *ED*, *BW* and *AT* are as defined earlier in Equation (1) above.

#### 3.1.3. Dermal Contact with Soil

(3) $$ADI_{dems}=\frac{C_{s}\times SA\times FE\times AF\times ABS\times EF\times ED\times CF}{BW\times AT}$$

where *ADI_dems_* is the exposure dose via dermal contact in mg/kg/day. *C_S_* is the concentration of heavy metal in soil in mg/kg, *SA* is exposed skin area in cm^2^, *FE* is the fraction of the dermal exposure ratio to soil, *AF* is the soil adherence factor in mg/cm^2^, *ABS* is the fraction of the applied dose absorbed across the skin. *EF*, *ED*, *BW*, *CF* and *AT* are as defined earlier in Equation (1) before. Table 1 shows the exposure parameters used for the health risk assessment for standard residential exposure scenario through different exposure pathways.

### 3.2. Non-Carcinogenic Risk Assessment

Non-carcinogenic hazards are characterized by a term called hazard quotient (*HQ*). *HQ* is a unitless number that is expressed as the probability of an individual suffering an adverse effect. It is defined as the quotient of *ADI* or dose divided by the toxicity threshold value, which is referred to as the chronic reference dose (*RfD*) in mg/kg-day of a specific heavy metal as shown in Equation (4) [22]:

(4) $$HQ=\frac{ADI}{RfD}$$

For *n* number of heavy metals, the non-carcinogenic effect to the population is as a result of the summation of all the *HQs* due to individual heavy metals. This is considered to be another term called the Hazard Index (*HI*) as described by USEPA document [22]. Equation (5) shows the mathematical representation of this parameter:

(5) $$HI=\overset{n}{\underset{k=1}{\sum }}HQ_{k}=\overset{n}{\underset{k=1}{\sum }}\frac{ADI_{k}}{RfD_{k}}$$

where *HQ_k_, ADI_k_* and *RfD_k_* are values of heavy metal *k.* If the *HI* value is less than one, the exposed population is unlikely to experience adverse health effects. If the *HI* value exceeds one, then there may be concern for potential non-carcinogenic effects [22].

### 3.3. Carcinogenic Risk Assessment

For carcinogens, the risks are estimated as the incremental probability of an individual developing cancer over a lifetime as a result of exposure to the potential carcinogen. The equation for calculating the excess lifetime cancer risk is:

(6) $$Risk_{pathway}=\overset{n}{\underset{k=1}{\sum }}ADI_{k}CSF_{k}$$

where *Risk* is a unitless probability of an individual developing cancer over a lifetime. *ADI_k_* (mg/kg/day) and *CSF_k_* (mg/kg/day)^−1^ are the average daily intake and the cancer slope factor, respectively for the kth heavy metal, for *n* number of heavy metals. The slope factor converts the estimated daily intake of the heavy metal averaged over a lifetime of exposure directly to incremental risk of an individual developing cancer [22].

The total excess lifetime cancer risk for an individual is finally calculated from the average contribution of the individual heavy metals for all the pathways using the following equation:

*Risk_(total)_ = Risk _(ing)_ + Risk _(inh)_ + Risk _(dermal)_* (7)

where *Risk _(ing)_*, *Risk _(inh)_*, and *Risk _(dermal)_* are risks contributions through ingestion, inhalation and dermal pathways.

Both non-carcinogenic and carcinogenic risk assessment of heavy metals are calculated using *RfD* and *CSF* values derived largely from the Department of Environmental Affairs (South Africa) and USEPA as shown in Table 2.

## 4. Results and Discussion

### 4.1. Concentrations of Heavy Metals in Soil from the Gold Mining Area

Average concentrations of heavy metals in mg/kg from the different locations of the gold mining area are presented in Table 3. The concentrations were used to calculate average daily intakes for non-carcinogenic and carcinogenic risk assessment.

The results presented showed that the average concentrations of the heavy metals in soil from the gold mining area varied significantly and decreased in the order of Cr > Ni > As > Zn > Cu > Co > Pb > Hg > Cd. The average ranges were as follows: Cr (77.50–861.67 mg/kg); Ni (68.33–152.50 mg/kg); As (65.17–115.19 mg/kg); Zn (21.82–82.50 mg/kg); Cu (19.09–55.83 mg/kg); Co (11.82–33.68 mg/kg); Pb (1.58–10.22 mg/kg); Hg (0.06–0.13 mg/kg); and Cd (0.04–0.05 mg/kg) respectively. It was also discovered that the minimum concentration of Cr (30.00 mg/kg) was recorded in Tailings three and a maximum of 1360.00 mg/kg from West Village. For As, the minimum concentration was 39.40 mg/kg in Tailings three and a maximum of 299.50 mg/kg from Tailings two. On the other hand, Ni recorded a minimum concentration of 10.00 mg/kg in the West village while a maximum of 220.00 mg/kg was recorded in Tailings three. Concentrations of Zn, Cu, Co, Pb, Hg, and Cd did not deviate much from the average values presented in Table 3. With all the heavy metals considered, results indicated that mine tailings had generally higher concentrations of heavy metals compared to the soil from mine villages.

Compared with recommended maximum allowable limits for South Africa and from other countries as shown in Table 4, As and Cr were found to be the highest in the present study. These highest levels of As and Cr could be linked to alleged sickness in children suffering from diarrheal diseases and chest pains. However, Pb, Cd, Zn, were lower than the maximum allowable limits while Hg, Cu, Co and Ni were comparable with other countries.

### 4.2. Non-Carcinogenic Risk of Heavy Metals for Adults and Children

**Figure 2:**
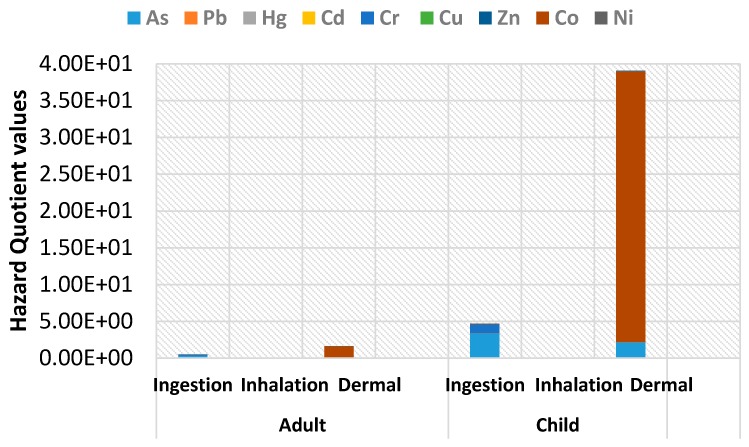
Hazard quotient (*HQ*) values for heavy metals in adults and children for soil from mining area.

Non carcinogenic risk for adults and children were calculated based *RfD* values as presented in Table 2 and *ADI* values in Table 5. These results for the ingestion, inhalation and dermal pathways are all presented in terms of *HQ*s as shown in Figure 2.

When *HQ* and *HI* values are less than 1, there is no obvious risk to the population, but if these values exceed one, there may be concern for potential non-carcinogenic effects [23]. For the adult population, calculated values of *HQ* were less than one in ingestion and inhalation pathways. However, *HI* for all the pathways was equal to 2.13, a value greater than one due to the dermal pathway. This meant that the adult population was at risk of non-carcinogenic effects. For children, the ingestion and dermal pathways had *HQ* and *HI* values greater than 1 mainly driven by Cr and As giving a total *HI* of 43.80 for all the pathways. This high value indicated heavy metal pollution that may pose a very high non cancer health risk to children living around the gold mining area. The results also indicate that, in both adults and children, the dermal pathway contributes the greatest to non-carcinogenic risk followed by the ingestion pathway. Inhalation is the least contributor to the risk.

### 4.3. Carcinogenic Risk Assessment of Heavy Metals for Adults and Children

**Figure 3:**
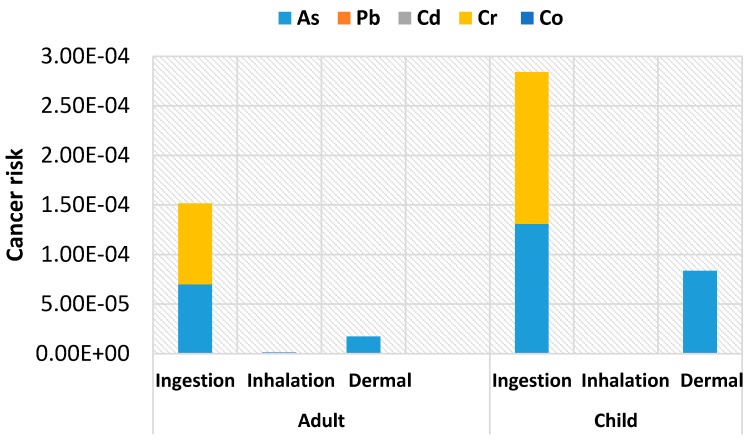
Cancer risk values of heavy metals for adults and children in soil from mining area.

The excess lifetime cancer risks for adults and children are calculated separately from the average contribution of the individual heavy metals in soil for all the pathways using Equations (6) and (7). Based on the carcinogenic risk values of the calculated *ADI* values presented in Table 6, the results of the excess lifetime cancer risks are presented in Figure 3.

The carcinogenic risk was calculated based on As, Pb, Cd, Cr and Co. As and Cr was found to be the highest contributors to the cancer risk. The US Environmental Protection Agency considers acceptable for regulatory purposes a cancer risk in the range of 1 × 10^−6^ to 1 × 10^−4^ [23]. On the other hand, South Africa, considers the Individual cancer risk limit to be 5 × 10^−6^ [38]. The cancer risk for adults was found to be 1.7 × 10^−4^ (1 in 5882 individuals) and 3.67 × 10^−4^ ( 1 in 2725 individuals) for children, which were both higher than acceptable values. In the study area, children are therefore more at risk than adults. The ingestion route seems to be the major contributor to excess lifetime cancer risk followed by the dermal pathway.

## 5. Conclusions

The results showed that the average concentrations of the heavy metals in soil from the gold mining area varied significantly and decreased in the order of Cr > Ni > As > Zn > Cu > Co > Pb > Hg > Cd. Compared with recommended maximum allowable limits from South Africa and other countries, Cr, As and Ni were found to be the highest. Cr was 43 times higher than the South African maximum allowable limit, while As was 14 times greater. On the other hand, Ni was 1.2 times higher than the South African maximum allowable limit. The results also indicated that, in both adults and children, the dermal pathway was the greatest contributor to the non-carcinogenic risk followed by the ingestion pathway. The inhalation pathway was the least contributor to non-cancer risk. For the carcinogenic effect, the ingestion pathway contributed the most to cancer risk followed by the dermal pathway Based on the results of this study, it can be concluded that soils surrounding the gold mining area are seriously polluted by heavy metals, especially from As, Cr and Ni. This quantitative evidence demonstrates the critical need to put in place mining regulations to protect residents, especially children from heavy metal pollution in the environment.

## Figures and Tables

**Table 1:** Exposure parameters used for the health risk assessment through different exposure pathways for soil.

Parameter	Unit	Child	Adult	References
Body weight (*BW*)	kg	15	70	[24]
Exposure frequency (*EF*)	days/year	350	350	[24]
Exposure duration (*ED*)	years	6	30	[24]
Ingestion rate (*IR*)	mg/day	200	100	[24]
Inhalation rate (*IRair*)	m^3^/day	10	20	[24]
Skin surface area (*SA*)	cm^2^	2100	5800	[24]
Soil adherence factor (*AF*)	mg/cm^2^	0.2	0.07	[24]
Dermal Absorption factor (*ABS*)	none	0.1	0.1	[24]
Dermal exposure ratio (*FE*)	none	0.61	0.61	[24]
Particulate emission factor (*PEF*)	m^3^/kg	1.3 × 10^9^	1.3 × 10^9^	[24]
Conversion factor (*CF*)	kg/mg	10^−6^	10^−6^	[23]
Average time (*AT*)	days			[24]
For carcinogens		365 × 70	365 × 70	[24]
For non-carcinogens		365 × ED	365 × ED	[24]

**Table 2:** Reference doses (*RfD*) in (mg/kg-day) and Cancer Slope Factors (*CSF*) for the different heavy metals.

Heavy Metal	Oral *RfD*	Dermal *RfD*	Inhalation *RfD*	Oral *CSF*	Dermal *CSF*	Inhalation *CSF*	References
As	3.00E−04	3.00E−04	3.00E−04	1.50E+00	1.50E+00	1.50E+01	[24,25]
Pb	3.60E−03	-	-	8.50E−03	-	4.20E−02	[24,26]
Hg	3.00E−04	3.00E−04	8.60E−05	-	-	-	[24]
Cd	5.00E−04	5.00E−04	5.70E−05	-	-	6.30E+00	[24,25]
Cr (VI)	3.00E−03	-	3.00E−05	5.00E−01	-	4.10E+01	[19,24]
Co	2.00E−02	5.70E−06	5.70E−06	-	-	9.80E+00	[27]
Ni	2.00E−02	5.60E−03	-	-	-	-	[24]
Cu	3.7.00E−02	2.40E−02	-	-	-	-	[24,27]
Zn	3.00E−01	7.50E−02	-	-	-	-	[24,27]

**Table 3:** Average concentrations (mg/kg) of heavy metals in in soil from the different mine locations.

Location & GPS Points	No. of Samples	Average Concentrations of Heavy Metals in Different Locations in mg·kg^−1^								
		As	Pb	Hg	Cd	Cr	Cu	Zn	Co	Ni
Tailings one (26°22’ S:27°29’ E)	11	94.17	8.85	0.13	0.05	441.52	46.78	46.15	33.68	131.04
Tailings two (26°22’ S:27°30’ E)	13	115.19	10.22	0.13	0.05	270.76	45.48	51.95	31.76	115.08
Tailings three (26°22’ S:27°26’ E)	8	71.33	2.31	0.06	0.05	77.50	46.25	82.50	30.00	152.50
Tailings four (26°21’ S:27°27’ E)	12	73.18	2.96	0.07	0.04	104.17	55.83	60.00	21.67	99.83
Tailings five (26°23’ S:27°25’ E)	12	67.08	3.31	0.06	0.05	97.50	47.50	48.33	21.67	125.83
West village (26°23’ S:27°28’ E)	6	65.17	1.58	0.10	0.05	861.67	36.67	48.33	28.33	68.33
East village (26°22’ S:27°30’ E)	11	69.69	4.32	0.06	0.05	98.18	19.09	21.82	11.82	91.82
Average		79.40	4.79	0.09	0.05	278.76	42.51	51.30	25.56	112.06
Minimum		65.17	1.58	0.06	0.04	77.50	19.09	21.82	11.82	68.33
Maximum		115.19	10.22	0.13	0.05	861.67	55.83	82.50	33.68	152.50

**Table 4:** Maximum allowable limit of heavy metals concentrations in soil (mg × kg^−1^) for different countries.

Country	Maximum Allowable Limit of Concentrations of Heavy Metals in Soil (mg × kg^−1^) for Different Countries									References
	As	Pb	Hg	Cd	Cr	Cu	Zn	Co	Ni	
Germany	50	70.0	0.5	1.0	60.0	40.0	150.0	n.a.	50.0	[28]
Poland	n.a.	100	n.a.	3	100	100	300	50	100	[29]
UK	32	450	10	10	130	n.a.	n.a.	n.a.	130	[30]
Australia	20	300	1	3	50	100	200	n.a.	60	[31]
Taiwan	60	300	2	5	250	200	600	n.a.	200	[28]
Bulgaria	10	26	0.03	0.4	65	34	88	20	46	[32]
Canada	20	200	0.8	3	250	150	500	n.a.	100	[33]
China	30	80	0.7	0.5	200	100	250	n.a.	50	[34]
Tanzania	1	200	2	1	100	200	150	n.a.	100	[35]
FAO/WHO Guidelines	20	100	n.a.	3	100	100	300	50	50	[36]
EU Guidelines	n.a.	300	n.a.	3	150	140	300	n.a.	75	[37]
South Africa	5.8	20	0.93	7.5	6.5	16	240	300	91	[24]

n.a.: Not available.

**Table 5:** Average daily intake (*ADI*) values in mg/kg/day for adults and children in soil from the mining area for non-carcinogenic risk calculations.

Receptor	Pathway	Average Daily Intake (*ADI*) Values for Heavy Metals in mg/kg/day									Total
		As	Pb	Hg	Cd	Cr	Cu	Zn	Co	Ni	
Adult	Ingestion	1.09E−04	6.57E−06	1.19E−07	6.61E−08	3.82E−04	5.82E−05	7.03E−05	3.50E−05	1.54E−04	8.14E−04
	Inhalation	1.60E−08	9.66E−10	1.75E−11	9.72E−12	5.62E−08	8.56E−09	1.03E−08	5.15E−09	2.26E−08	1.20E−07
	Dermal	2.69E−05	1.63E−06	2.95E−08	1.64E−08	9.46E−05	1.44E−05	1.74E−05	8.67E−06	3.80E−05	2.02E−04
	Total	1.36E−04	8.19E−06	1.49E−07	8.25E−08	4.76E−04	7.27E−05	8.77E−05	4.37E−05	1.92E−04	1.02E−03
Child	Ingestion	1.02E−03	6.13E−05	1.11E−06	6.17E−07	3.56E−03	5.44E−04	6.56E−04	3.27E−04	1.43E−03	7.60E−03
	Inhalation	4.07E−08	2.46E−09	4.46E−11	2.47E−11	1.43E−07	2.18E−08	2.63E−08	1.31E−08	5.74E−08	3.05E−07
	Dermal	6.50E−04	3.92E−05	7.13E−07	3.95E−07	2.28E−03	3.48E−04	4.20E−04	2.09E−04	9.18E−04	4.87E−03
	Total	1.67E−03	1.01E−04	1.83E−06	1.01E−06	5.85E−03	8.92E−04	1.08E−03	5.36E−04	2.35E−03	1.25E−02

**Table 6:** Average daily intake (*ADI*) values in mg/kg/day for adults and children in soil from the mining area for carcinogenic risk calculations.

Receptor	Pathway	Average Daily Intake (*ADI*) Values for Heavy Metals in mg/kg/day									Total
		As	Pb	Hg	Cd	Cr	Cu	Zn	Co	Ni	
Adult	Ingestion	4.66E−05	2.81E−06	5.11E−08	2.83E−08	1.64E−04	2.50E−05	3.01E−05	1.50E−05	6.58E−05	3.49E−04
	Inhalation	6.86E−09	4.14E−10	7.51E−12	4.17E−12	2.41E−08	3.67E−09	4.43E−09	2.21E−09	9.67E−09	5.13E−08
	Dermal	1.15E−05	6.97E−07	1.27E−08	7.02E−09	4.05E−05	6.18E−06	7.46E−06	3.72E−06	1.63E−05	8.64E−05
	Total	5.82E−05	3.51E−06	6.38E−08	3.54E−08	2.04E−04	3.11E−05	3.76E−05	1.87E−05	8.21E−05	4.36E−04
Child	Ingestion	8.70E−05	5.25E−06	9.54E−08	5.29E−08	3.05E−04	4.66E−05	5.62E−05	2.80E−05	1.23E−04	6.52E−04
	Inhalation	3.49E−09	2.10E−10	3.82E−12	2.12E−12	1.22E−08	1.87E−09	2.25E−09	1.12E−09	4.92E−09	2.61E−08
	Dermal	5.57E−05	3.36E−06	6.11E−08	3.39E−08	1.96E−04	2.98E−05	3.60E−05	1.79E−05	7.87E−05	4.17E−04
	Total	1.43E−04	8.62E−06	1.56E−07	8.68E−08	5.01E−04	7.64E−05	9.22E−05	4.60E−05	2.01E−04	1.07E−03
